# Unraveling the formation mechanism of 3-methylindole from tryptophan in the glucose-mediated Maillard reaction: a combined ReaxFF MD, DFT, and experimental study

**DOI:** 10.1016/j.fochx.2026.104719

**Published:** 2026-10-31

**Authors:** Yuan-Yuan Yang, Chao-Kun Wei, Xin Pan, An-Ran Zheng, Wen-Li Wang, Chun-Mei Yang, Yuan Liu

**Affiliations:** a School of Food Science and Engineering, Ningxia University, Yinchuan 750021, People's Republic of China; b School of Biological Science and Engineering, North Minzu University, Yinchuan 750021, People's Republic of China; c School of Agriculture & Biology, Shanghai Jiao Tong University, Shanghai 200240, People's Republic of China; d Ningxia Ningyang Food Co., Ltd., Wuzhong 751100, People's Republic of China

**Keywords:** Maillard reaction, Tryptophan, 3-Methylindole, ReaxFF MD, DFT

## Abstract

3-Methylindole is a concentration-dependent aroma compound, and its formation mechanism in the tryptophan-glucose Maillard reaction is crucial for flavor regulation but remains incompletely elucidated. Using gas chromatography-olfactometry-mass spectrometry (GC-O-MS), 3-methylindole was identified as a key odor-active compound (OAV > 1). Reactive molecular dynamics (ReaxFF MD) and density functional theory (DFT) revealed a sequential pathway involving nucleophilic addition, Amadori rearrangement, dehydration, decarboxylation, and Strecker degradation, and characterized 16 transition states and their activation energies, indicating a net endothermic reaction. Pyrolysis-gas chromatography–mass spectrometry (Py-GC–MS) detected methylglyoxal and indole-3-acetaldehyde as key intermediates, whose temperature-dependent concentration trends supported the proposed pathway. Differential scanning calorimetry (DSC) further confirmed the multi-stage endothermic behavior, consistent with the DFT results. This study proposes a complete formation mechanism of 3-methylindole, providing a theoretical basis for indole-type flavor generation and a methodological framework for studying volatile compounds in complex thermally processed food systems.

## 1 Introduction

The Maillard reaction, a non-enzymatic browning process initiated by the condensation of reducing sugars and amino compounds under thermal conditions, represents one of the most complex and influential reaction pathways in food chemistry. This cascade of transformations is responsible for generating a vast array of volatile compounds that define the aroma, taste, and color of thermally processed foods, from baked goods and roasted meats to coffee and fermented products (Cui et al., 2021). Beyond its desirable sensory contributions, the Maillard reaction also impacts nutritional quality by degrading essential amino acids and can lead to the formation of potentially detrimental compounds, highlighting the need for a precise understanding of its mechanistic pathways to better control food quality and safety (Shakoor et al., 2022).

Among amino acid precursors, tryptophan—an essential aromatic amino acid abundant in legumes—holds particular significance due to its unique indole ring structure (Alcázar-Valle et al., 2022; Ding et al., 2025; Sun et al., 2024). This structure not only underpins important physiological functions but also serves as the backbone for a variety of potent flavor-active indole derivatives formed during thermal degradation (Li et al., 2023; Liu et al., 2023). In the presence of reducing sugars such as glucose, tryptophan participates vigorously in Maillard chemistry, yielding products like indole, 2,3-dimethylindole, and notably, 3-methylindole. These compounds exhibit a remarkable duality in sensory impact: at low concentrations, they impart pleasant floral and jasmine-like notes, whereas at higher levels, they can contribute undesirable animal-like or fecal odors (Zou et al., 2024). Consequently, the controlled formation of these indoles is of great interest for flavor engineering in foods such as fermented sauces, dairy products, and alcoholic beverages, where 3-methylindole has been identified as a key aroma compound (Guan et al., 2024; Tian et al., 2024; Tang et al., 2022).

Despite its documented role in food flavor, the detailed chemical pathway through which 3-methylindole arises from the Maillard reaction between tryptophan and glucose remains largely speculative. Existing literature has extensively explored the biological metabolism of tryptophan in gut microbiota and its physiological implications (Zhao et al., 2025; Huang et al., 2026; Dong et al., 2026), yet the analogous abiotic reaction network under food processing conditions is comparatively uncharted. The inherent complexity of the Maillard system—with its myriad competing reactions, unstable intermediates, and sensitivity to parameters like temperature, pH, and reactant ratio—makes experimental isolation of mechanistic steps exceedingly challenging. This gap underscores the need for a sophisticated, multi-pronged investigative approach (Liu et al., 2026).

Advances in computational chemistry now provide powerful tools to probe such complex reaction systems at an atomic level. ReaxFF MD enables the simulation of bond-breaking and bond-forming events in condensed-phase systems under realistic time scales, offering a dynamic, macroscopic view of reaction pathways and intermediate formation (Sun et al., 2023). Complementarily, DFT delivers quantum-mechanical precision, allowing for the optimization of molecular geometries, the identification and validation of transition states, and the calculation of activation energies and thermodynamic parameters (Grambow et al., 2020). The synergy of these methods has been successfully demonstrated in elucidating pyrolysis mechanisms and reaction networks in diverse systems, from catalytic biomass decomposition to the oxidation of flavor aldehydes, as well as food-related fields such as seasonings (Cheng et al., 2022; Pan et al., 2026; Tian et al., 2026; Wang et al., 2024). In the food chemistry domain, the combined application of ReaxFF MD and DFT has enabled the mechanistic elucidation of flavor evolution during alcoholic beverage aging (Huang et al., 2026), the molecular-level characterization of multifunctional food additives (Anjum et al., 2026), the identification of competitive pathways in lipid oxidation (Xiao et al., 2024), and the mechanistic mapping of indole pyrolysis to nitrogen-containing species (Liu et al., 2024). However, despite these contributions, the formation mechanism of indole-type aroma compounds within the Maillard reaction network has remained largely unexplored. When integrated with modern analytical techniques such as GC-O-MS and Py-GC–MS, this combined approach forms a robust framework for connecting computational predictions with empirical observation.

Therefore, the objective of this study was to fully elucidate the formation mechanism of 3-methylindole in the tryptophan-glucose Maillard system by employing an integrated methodology. First, GC-O-MS is used to identify and prioritize key odor-active volatiles. Second, ReaxFF MD simulations are conducted to map potential reaction pathways and identify critical intermediates under simulated thermal processing conditions. Third, DFT calculations are applied to refine these pathways, locate transition states, and quantify energy barriers. Finally, targeted Py-GC–MS analysis is employed to experimentally detect the presence of key intermediates predicted by the simulations, providing fragment-level structural information that complements the computational findings. Furthermore, DSC experiments were employed to conduct a comprehensive analysis of the thermal behavior of the reaction system from a macroscopic thermodynamic perspective. By bridging multi-scale computational modeling with experimental validation, this work not only clarifies a specific flavor-formation mechanism but also establishes a transferable research framework for mechanistically studying volatile compound generation in complex food systems, ultimately contributing to the science-based design and control of food flavors.

## 2 Materials and methods

### 2.1 Material

*L*-tryptophan (analytical grade), d-glucose (analytical grade), 2-Methyl-3-heptanone standard (chromatography grade), 2,5-dimethylpyrazine (> 98%), indole (> 98%), 3-methylindole (> 99%), 5-methyl-2-furanmethanol (> 97%), ethyl caproate (> 99%), octanal (> 99%), nonanal (> 96%), decanal (> 97%), 2-acetylpyrrole (> 98%), quinoline (> 98%) purchased from McLean Company (Shanghai, China); Sodium dihydrogen phosphate (analytical grade), disodium hydrogen phosphate (analytical grade), methanol (chromatographic grade) purchased from Beijing Chemical Reagent Company;n-alkanes (C_7_–C_30_); Sodium hydroxide (analytical grade), hydrochloric acid (analytical grade), acetonitrile (chromatography grade), formic acid (chromatography grade) were purchased from Sinopharm Chemical Reagent Co., Ltd.

### 2.2 Preparation of MRPs

Tryptophan (0.4 g) and Glucose (0.6 g) were dissolved in 10 mL of phosphate buffer (pH 6, 0.2 mol/L), transferred to a 20 mL pressure-resistant flask, and react at 120, 130, and 140 °C under pH 6 conditions for 1.5 h to obtain Maillard reaction products and stored at −80 °C for subsequent experiments.

### 2.3 GC-O analysis method

After GC separation, the samples entered both the mass spectrometer detector and the olfactory detector with a split ratio of 1:1. The sniff port temperature was maintained at 150 °C. Moist air was injected into the sniff port using nitrogen (99.99%) as the carrier gas to prevent drying of the evaluator's nasal mucosa, reduce olfactory fatigue, and maintain olfactory sensitivity.

The odor analysis panel comprised five trained evaluators (three males and two females). Each evaluator independently assessed the samples, recording perceived odor characteristics and intensity levels, with results averaged. Rating scale: 1 = weak, 3 = moderate, 5 = strong (Zhu et al., 2022).

### 2.4 GC–MS analysis method

An aliquot of 2 mL of the sample was transferred to a 20 mL headspace vial, and 1 μL of 2-methyl-3-heptanone internal standard (50 μg/mL in methanol) was added. Solid-phase microextraction (SPME) was then performed on each sample. The sample was equilibrated at 60 °C for 10 min, followed by headspace adsorption for 20 min prior to injection. The injection temperature was set at 250 °C, with a desorption time of 5 min. Helium was used as the carrier gas at a flow rate of 1.0 mL/min. The oven temperature program was as follows: held at 40 °C for 2 min, ramped from 40 to 80 °C at 3 °C/min and held for 3 min, then ramped from 80 to 120 °C at 4 °C/min and held for 2 min, and finally ramped from 120 to 230 °C at 10 °C/min. Splitless injection mode was employed. The ion source temperature was maintained at 230 °C, with an electron energy of 70 eV, a quadrupole temperature of 150 °C, and a mass scan range of *m*/*z* 40–450. Volatile compounds were qualitatively identified by comparing their mass spectra with the NIST17 and NIST17s databases, and only compounds with a matching similarity greater than 80% were considered positively identified. Semi-quantitative analysis of volatile organic compounds (VOCs) was carried out using the internal standard method according to the formula Cv = (Sv/Si) × Ci, where Cv is the concentration of the volatile compound, Ci is the concentration of the internal standard, Sv is the peak area of the volatile compound, and Si is the peak area of the internal standard.

### 2.5 ReaxFF MD

Single-molecule models of tryptophan and glucose were constructed using Materials Studio software, and geometric optimization was performed in the Dmol3 module. A multi-molecule mixture model was then built from the optimized single-molecule models using the Amorphous Cell (AC) module. The molar ratio of tryptophan to glucose (C₁₁H₁₂N₂O₂/C₆H₁₂O₆) was set to approximately 29:50. Accordingly, 29 tryptophan molecules and 50 glucose molecules were selected to establish the tryptophan–glucose Maillard reaction model. To obtain a stable initial configuration, the system was relaxed under NVT ensemble at 1000 K for 40 ps with energy minimization using the conjugate gradient method. Reactive simulations were then performed under NPT ensemble at three temperatures (2100, 2400, 2700 K) and two pressures (100, 150 MPa). Elevated temperatures were employed to accelerate reactions within the limited simulation timescale, while elevated pressures were included to assess confinement effects on the reactive system (Liu et al., 2026). The initial density of the simulation box was set to 0.2 g/cm^3^, with a cubic box dimension of 49.9831 × 49.9831 × 49.9831 Å^3^. To obtain a stable initial configuration, Total simulation time was 1000 ps with a timestep of 0.25 fs, and trajectories were recorded every 5 ps. Temperature and pressure were controlled by the Berendsen thermostat and barostat (damping constant: 100 fs). Chemical events were identified based on dynamic bond orders (cutoff: 0.3). All simulations used the LAMMPS package, and trajectories were visualized with OVITO software. The constructed model is shown in Fig. 1, where C, H, N, and O atoms are represented by gray, white, blue, and red, respectively. Simulation results were visualized using OVITO software to observe the motion state of the entire simulated system, thereby investigating the formation pathway of 3-methylindole in the tryptophan-glucose Maillard reaction system (Sun et al., 2023; Zhu et al., 2022).

**Fig. 1:**
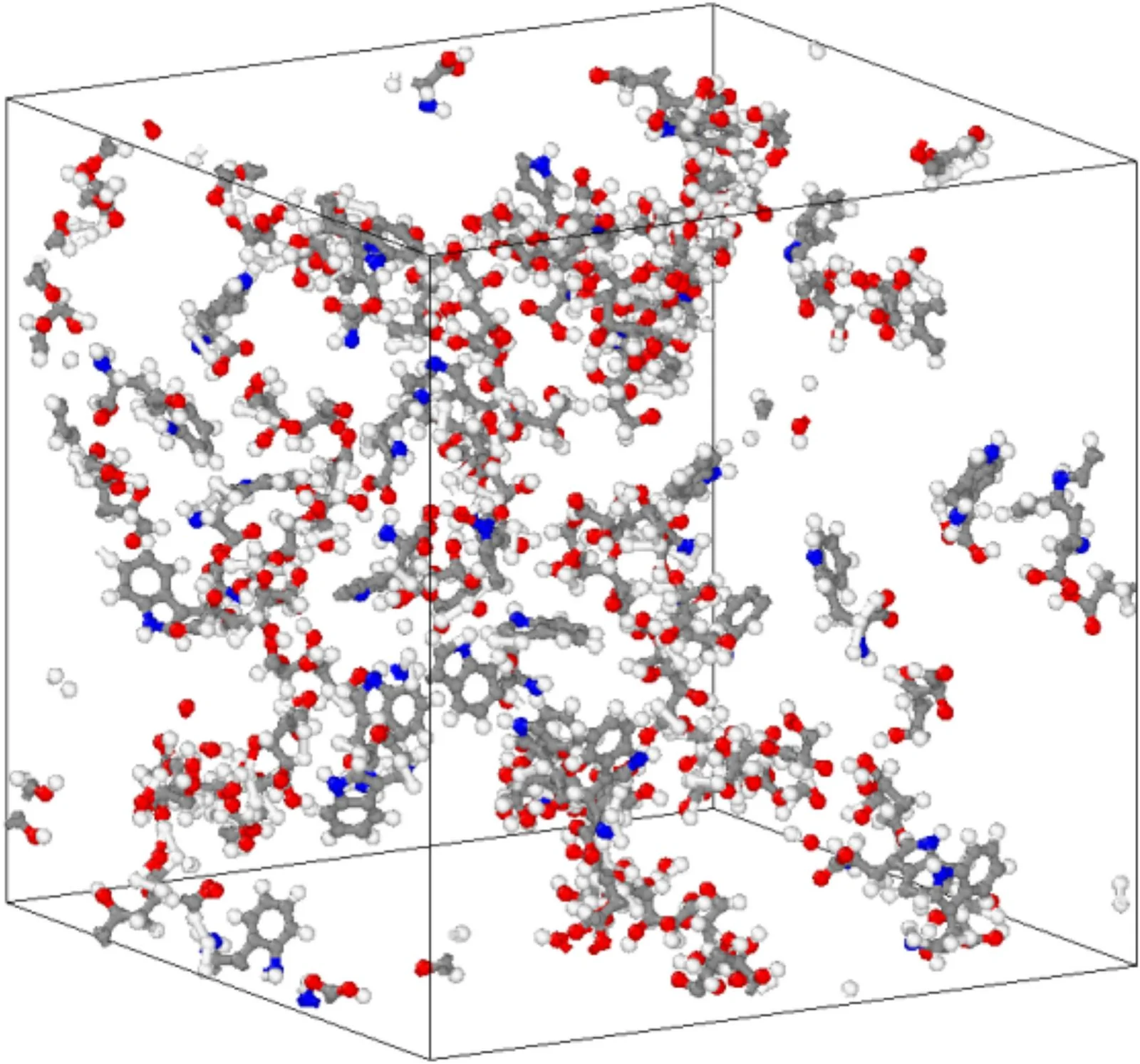
The 3D model of the multi-molecular mixture of Tryptophan-Glucose Maillard product. Among them, the gray, white, blue and red subtables represent C, H, N and O atoms. (For interpretation of the references to color in this figure legend, the reader is referred to the web version of this article.)

### 2.6 DFT calculations

Based on the reaction pathways obtained from molecular dynamics simulations, structural optimizations and frequency calculations were performed on reactants, intermediates, and products during the 3-methylindole formation process using the B3LYP/6-311G+(d,p) basis set within the Gaussian 16 quantum chemistry package. This ensured the absence of virtual frequencies for subsequent transition state searches. Transition states along the entire reaction pathway were searched using a combination of TS, QST2, and QST3 methods. First, the vibrational directions of transition states were examined. Second, the correctness of transition states was verified using IRC intrinsic reaction coordinates. Finally, the energy barriers for the entire reaction process were calculated to observe the energy changes during the thermally induced formation of 3-methylindole from tryptophan-glucose (Anjum et al., 2026; Zhang et al., 2023; Zheng et al., 2024).

### 2.7 Py-GC/MS

Sample preparation followed the GC-O-MS procedure. Py-GC/MS employs automated sample introduction: weighed samples are placed into hollow quartz tubes, sealed at both ends with quartz wool, and positioned within the pyrolyzer's heating coil. Samples undergo rapid heating at a rate of 10 °C/ms to 550 °C, where they are held for 15 s. Pyrolyzed volatiles are carried by helium carrier gas through the transfer line into the GC/MS. To prevent condensation of pyrolyzed volatiles before entering the GC/MS, the thermal valve and transfer line are maintained at elevated temperatures of 290 °C and 280 °C, respectively. Finally, pyrolysis products are introduced into the GC–MS for analysis. The GC/MS injection port temperature is 280 °C with a 1 μL injection volume. The temperature ramp program is: 50 °C held for 2 min, then ramped at 5 °C/min to 280 °C and held for 20 min. The split ratio is 25:1. Finally, the ion source temperature was set at 230 °C, ionization energy at 70 eV, transfer line temperature at 280 °C, and mass scan range from 30 to 550 (*m*/*z*). Peaks matching NIST17 and NIST17s databases based on peak area percentages were manually confirmed, followed by semi-quantitative analysis (Cheng et al., 2022).

### 2.8 DSC experiments

To investigate the thermal reaction properties of the system, DSC was employed to analyze its heat evolution. Single samples of tryptophan and glucose, as well as physical mixture powders, were taken; approximately 5.0 mg of each sample was weighed and heated from 0 °C to 500 °C at a rate of 10 °C/min under a high-purity nitrogen atmosphere (50 mL/min), with the DSC curve recorded throughout. (Zhong et al., 2019).

### 2.9 Statistical analysis of data

Statistical analysis of one-way ANOVA using SPSS Statistics 20.0 indicates a significance level of *p* < 0.05. All experimental data are expressed as mean ± standard deviation, with three replicates per group.

## 3 Results and discussion

### 3.1 Analysis of volatile components in the Maillard reaction of tryptophan-glucose

The composition of volatile substances in the tryptophan-glucose Maillard reaction system was investigated at three temperatures (120, 130, and 140 °C) using GC-O-MS. As expected, temperature significantly influenced the reaction profile; higher temperatures generally increased the variety and concentration of volatile flavor compounds (Guo et al., 2022). A total of 28 volatile compounds were identified and categorized (see Appendix Table 1). These compounds were categorized based on functional groups and structures into: 8 aldehydes, 7 ketones, 2 furans, 2 indoles, etc. Consistent with the unique indole ring structure of tryptophan, indole-type products were generated in notable concentrations under thermal conditions (Fiore et al., 2021). Additionally, compounds such as 2,5-dimethylpyrazine, 5-methyl-2-furancarboxaldehyde, 1-nonanal, and 2-acetylpyrrole were identified, contributing to fruity, grassy, caramel, and baked aromas (Gao et al., 2025; Wang et al., 2024; Zhang et al., 2020; Zhou et al., 2024). Importantly, products of the Strecker degradation, such as glyoxal and methylglyoxal, were also identified, providing initial observational clues for the reaction pathway leading to smaller flavor molecules (Ahn et al., 2025; Deng et al., 2025).

OAVs were used to characterize the contribution of aroma compounds to the overall flavor (Chen et al., 2025). Ten compounds with OAV > 1 were selected as key contributors (Table 1). The identities of all ten key aroma compounds (OAV > 1) were confirmed by pure standards. During olfactory assessment, indole and 3-methylindole exhibited pronounced jasmine-like aromas (Zhang et al., 2025). while 1-nonanal presented rich fruity notes (Zhang et al., 2020). These three flavor compounds constitute the pleasant odors generated in the thermally induced reaction of tryptophan. (Frøst et al., 2021), Among these, 3-methylindole showed the highest concentration range (16.87–21.2 μg/kg) and a maximum odor intensity rating of 5 (strong), establishing it as the primary compound for subsequent mechanistic investigation.

**Table 1:** The key volatile substances in the tryptophan-glucose Maillard reaction system identified by GC-O-MS and OAV > 1.

	Volatile compounds	CAS	Odor description^a^	Threshold/(mg/kg)	OAV/(μg/kg)^b^			Odor intensity^c^
					120 °C	130 °C	140 °C	
1	2,5-Dimethyl-pyrazine	123-32-0	Baking aroma, nut aroma	0.718	1.06	1.35	2.05	4
2	5-Methyl-2-furanmethanol	3857-25-8	Caramel flavor	0.1	26.5	28.5	26.1	2
3	Ethyl ester hexanoic acid	123-66-0	Fragrance, chemical flavor	0.005	50	54	70	4
4	Octanal	124-13-0	Grassy, citrus aromas	0.069	5.36	6.09	4.2	2
5	Nonanal	124-19-6	Flowery, aroma	0.008	155	167.5	158.75	3
6	Decanal	112-31-2	Bitter taste, lemon flavor	0.03	27.67	41.33	38	3
7	2-Acetylpyrrole	1072-83-9	Baking aroma	0.01	24	34	50	4
8	Quinoline	91-22-5	Oily flavor	0.71	2.03	2.2	2.28	3
9	Indole	120-72-9	Jasmine fragrance	0.04	42	47.5	45	5
10	3-Methyl-indole	83-34-1	Cream flavor, jasmine fragrance	0.15	16.87	21.2	19.2	5

a The Odor characteristic of each volatile compound sensed on sniffer port during GC-O-MS experiments.

b OAV = substance content/substance threshold.OAV ≥ 1 contributes more to the overall flavor, and the greater the OAV value of the volatiles, the greater the impact on the overall flavor. The odor threshold data used for OAV calculations come from published literature and related food flavor databases (Liu et al., 2014).

The Odor characteristic of each volatile compound sensed on sniffer port during GC-O-MS experiments.

c Mean odor intensity (0–5 scale, five assessors). In case compounds were not detected by all assessors, the number of those who positively identified the compound is indicated in parentheses.

Previous studies have identified indole-related compounds in fermented foods and examined their association with aromatic amino acid metabolism (Guan et al., 2024; Tang et al., 2022; Tian et al., 2024). Such observations establish their relevance to food aroma but do not resolve the non-biological formation chemistry operating in a heated tryptophan–glucose system. The present results provide a starting point for investigating a thermal formation route distinct from microbial metabolism. Moreover, odor activity in this model system should not be interpreted as evidence of a universally desirable sensory contribution. The concentration-dependent sensory behavior of indole compounds underscores the need to understand their formation and subsequent transformation during processing (Zou et al., 2024).

### 3.2 Pyrolytic analysis of the tryptophan-glucose Maillard reaction system

ReaxFF MD simulations were employed to model the complete pyrolysis pathway of the tryptophan-glucose system at the atomic scale. The simulations, conducted at different temperatures (2100, 2400, 2700 K) and pressures (100, 150 MPa), tracked the evolution of products and energy systems. ReaxFF MD simulations enabled the modeling of the complete reaction pathway from initial reactants to final products, thereby elucidating key intermediates in the Maillard reaction, complex pathways, and kinetic parameters at the atomic scale to reveal reaction mechanisms.

The number of pyrolysis products showed a trend of initial increase followed by stabilization, indicating reaction completion (Fig. 2a-b) (Wang, Jiang, & Luo, 2023). The evolution of the system's potential energy (PE) effectively reflects its approach to equilibrium (Sun et al., 2023). As shown in Fig. 2c, the PE increased over time, peaking near 800 ps before stabilizing. The final PE value increased with rising temperature, indicating that elevated temperatures drive the reaction system forward. Conversely, increasing pressure reduced the system density and the overall potential energy, making the reaction less favorable (Fig. 2d).

**Fig. 2:**
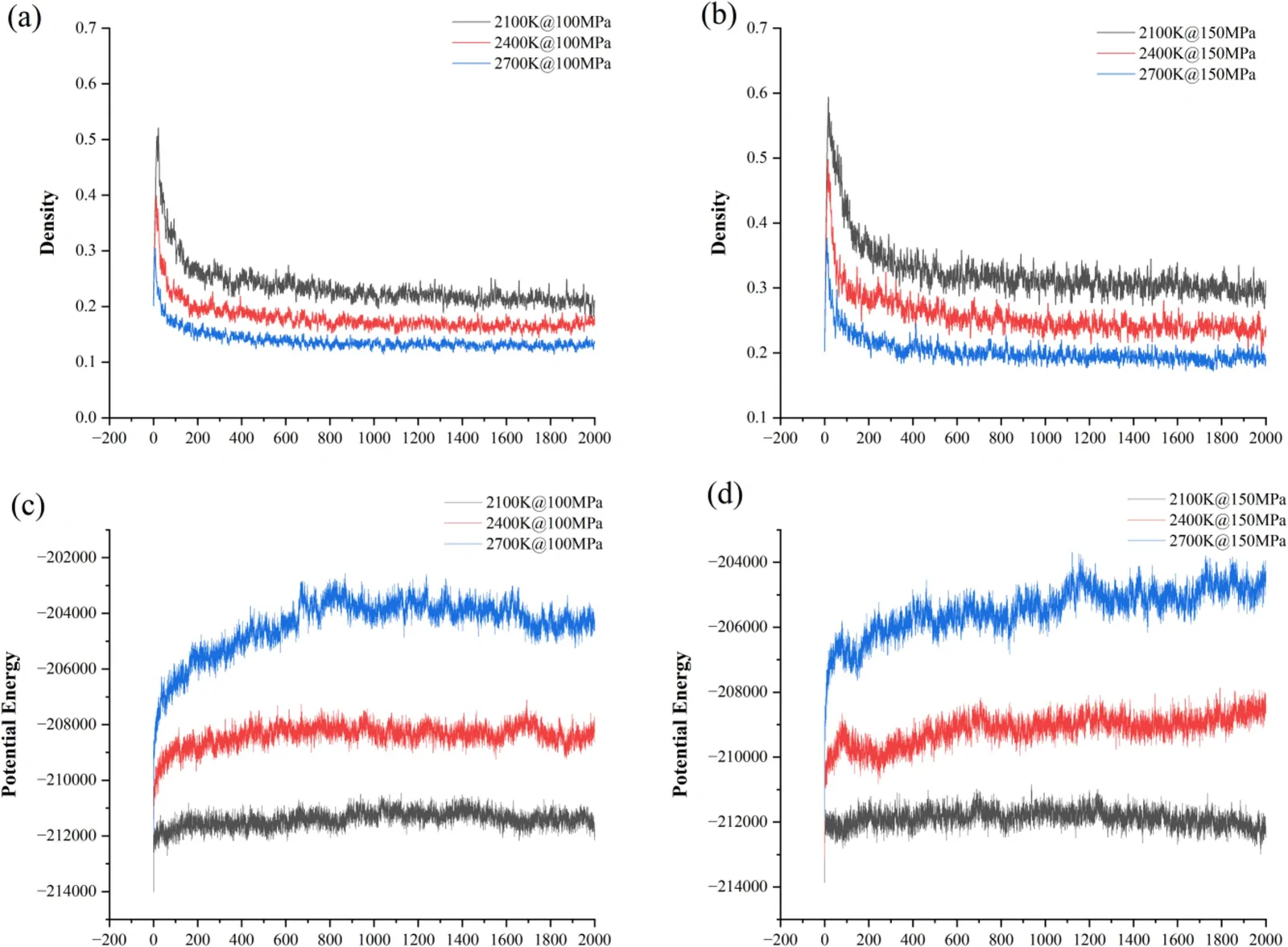
Evolution of potential energy and density of tryptophan-glucose system with pressure (100 MPa, 150 MPa) at different temperatures.

The simulations tracked the formation of small molecule intermediates, including H₂O, CO₂, formaldehyde, and the critical α-diketones, glyoxal and methylglyoxal (Table 2). Among these, α-diketals are produced by the cleavage of desoxyhexose, which influences the subsequent Strecker degradation of tryptophan to form indole aldehyde. As temperature increases, the onset time for glycol and methylglycol shortens while their quantities rise, indicating that elevated temperatures promote product formation. Concurrently, high temperatures accelerate the decomposition of H₂O into hydrogen-containing compounds, leading to increased quantities of most hydrogen-containing intermediates with rising temperature (Sun et al., 2023). Under conditions of 2100 K/100 MPa, methyl glyoxal appears at 218 ps with a maximum yield of 8; whereas under conditions of 2700 K/100 MPa, its appearance time is shortened to 14 ps. This temperature-dependent behavior validates the role of methyl glyoxal as a key intermediate in the proposed reaction pathway. However, pressure inhibits the formation of various substances. At the same temperature, increasing pressure leads to a decreasing trend in substance quantities. Compared to pressure, temperature exerts a more significant influence on the entire system. Therefore, in subsequent Py-GC/MS analyses, this study focuses on elucidating the effect of temperature on the tryptophan-glucose Maillard reaction system.

**Table 2:** Evolution details of various species.

*Species*	*Appearance time (ps)*						*Max numbers*					
	2100 K		2400 K		2700 K		2100 K		2400 K		2700 K	
	100 MPa	150 MPa	100 MPa	150 MPa	100 MPa	150 MPa	100 MPa	150 MPa	100 MPa	150 MPa	100 MPa	150 MPa
CH_4_	1754	1282	580	766	204	58	1	3	2	2	7	5
H_2_O	2	2	2	2	2	2	127	110	142	137	121	127
CO_2_	20	10	8	4	2	4	39	42	46	43	60	52
CH_3_	78	712	278	252	144	30	3	3	2	44	6	5
CH_3_O	30	46	2	10	4	4	3	3	2	45	5	7
CH_2_O	30	34	8	14	2	4	20	26	25	46	25	32
C_3_H_3_	26	544	54	136	14	24	1	2	3	47	7	7
HO	14	4	4	4	2	2	3	3	3	48	5	4
H_2_	120	26	20	30	8	12	7	4	20	49	44	30
C_2_H_4_	144	176	166	250	262	28	2	0	5	50	16	12
C_2_H_2_	36	22	62	58	16	24	18	17	30	51	67	55
C_2_H_2_O_2_	52	68	12	10	6	8	31	22	60	52	63	58
H_3_N	5	14	34	18	14	2	8	16	19	53	19	24
C_3_H_4_O_2_	218	48	40	78	14	16	8	6	54	9	21	15
H_2_N	ND	ND	ND	ND	ND	2	0	0	0	55	0	7

### 3.3 Formation pathway of 3-Methylindole

ReaxFF MD simulations elucidated a multi-step formation pathway for 3-methylindole, Molecular motions between tryptophan and glucose were observed by combining OVITO visualization with the ReacNetGenerator reaction network (Zeng et al., 2020). The proposed pathway links the established Maillard reaction concept to the conversion of tryptophan side chains. At present, studies have investigated the formation and degradation of Amadori products and the formation of aroma compounds in amino acid-sugar systems (Cui et al., 2021; Gao et al., 2025; Lei et al., 2024). On this basis, this study used ReaxFF MD simulation to connect these continuous reaction stages into a complete pathway from tryptophan and glucose to 3-methylindole, rather than just focusing on single reactions such as Amadori rearrangement or Strecker degradation.

This pathway primarily involves dehydration dehydration, enolization, decarboxylation, hydrogen transfer, and Strecker degradation reactions between tryptophan and glucose (Fig. 3). The key process begins with the nucleophilic addition of the amino group of tryptophan to the carbonyl group of glucose. This is followed by Amadori rearrangement and enol isomerization (Lei et al., 2024). Subsequent 2,3-enolization of the ARP yields 1-deoxythreose (Wang et al., 2026), After intramolecular hydrogen transfer and elimination of one molecule of C_3_H_6_O_3_, 1-deoxythreose yields methylmalonaldehyde. Finally, tryptophan undergoes Strecker degradation with methylglyoxal to form indole-3-acetaldehyde. The amino group of tryptophan undergoes nucleophilic addition to the aldehyde group of methylglyoxal, releasing one molecule of H_2_O to yield C_14_H_14_N_2_O_3_. Subsequently, C_14_H_14_N_2_O_3_ loses one molecule of CO_2_ to form C_13_H_14_N_2_O. After undergoing intramolecular hydrogen transfer, C_13_H_14_N_2_O reacts with H_2_O to yield C_13_H_16_N_2_O_2_. The C2 hydrogen of C_13_H_16_N_2_O_2_ transfers to the C1 position while undergoing a homolytic cleavage reaction, eliminating C_7_H_5_NO to yield indole-3-acetaldehyde. The final step is a decarbonylation of indole-3-acetaldehyde to produce 3-methylindole.

**Fig. 3:**
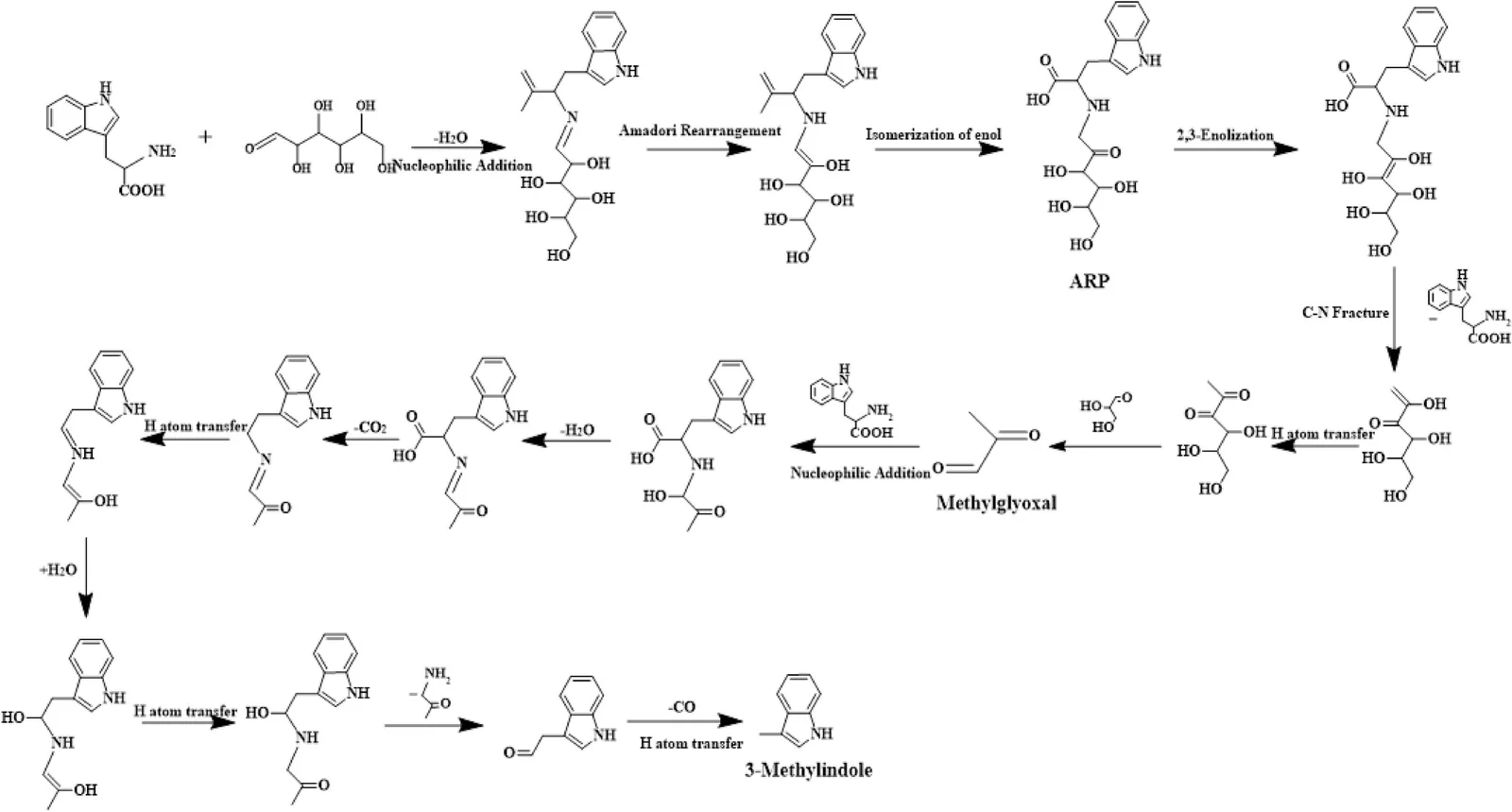
The formation mechanism of 3-methylindole in tryptophan-glucose Maillard reaction model system.

ReaxFF MD simulation of the tryptophan-glucose Maillard reaction to generate 3-methylindole provides a complete picture of the dynamic process, showing the specific evolution steps from the initial condensation to the formation of indole ring. To further explore the feasibility of the path, the DFT method is used to optimize and calculate the energy of the key intermediates and transition states in the path.

### 3.4 Transition state search and structure optimization of the 3-methylindole formation pathway

ReaxFF MD can simulate molecular/atomic behavior at the atomic scale, reproducing atomic trajectories during chemical reactions (Dufour-Décieux et al., 2021). However, the ReaxFF method relies on empirical potential functions, resulting in limited energy accuracy. Therefore, we further employ DFT calculations to refine the trajectories identified by ReaxFF: we perform geometric optimization, transition state search, and energy barrier calculations on the key intermediates and reaction steps observed along these ReaxFF trajectories.

Transition state theory posits that chemical reactions are not merely simple molecular collisions, but involve a transient high-energy transition state. This unstable state exists at the peak of critical energy, where chemical bonds partially break and reform. Ultimately, they may recombine into reactants or proceed to form products (Barrales-Martinez et al., 2024). DFT calculations were performed to refine the mechanism and characterize the transition states. Sixteen transition states (TS1-TS16) were successfully identified and validated. The validity of these transition states was preliminarily verified by analyzing their configurations and vibrational directions (Fig. 4a) (Otomo et al., 2021), which showed atomic motions oriented toward product formation. The vibrational directions of the transition states in the Fig indicate that atomic vibrations tend toward the product formation direction, while their configurations simultaneously exhibit characteristics of both reactants and products. The structure and vibrational modes of the transition states further reveal that the vibrational direction is a key factor promoting the transition state's conversion into the product. Further confirmation was obtained through Intrinsic Reaction Coordinate (IRC) analysis (Fig. S1), which traced the minimum energy path connecting reactants, transition states, and products, confirming each TS as the highest-energy point along its respective reaction coordinate (Barrales-Martinez et al., 2024; Wang, Wang & Wang, 2023).

**Fig. 4:**
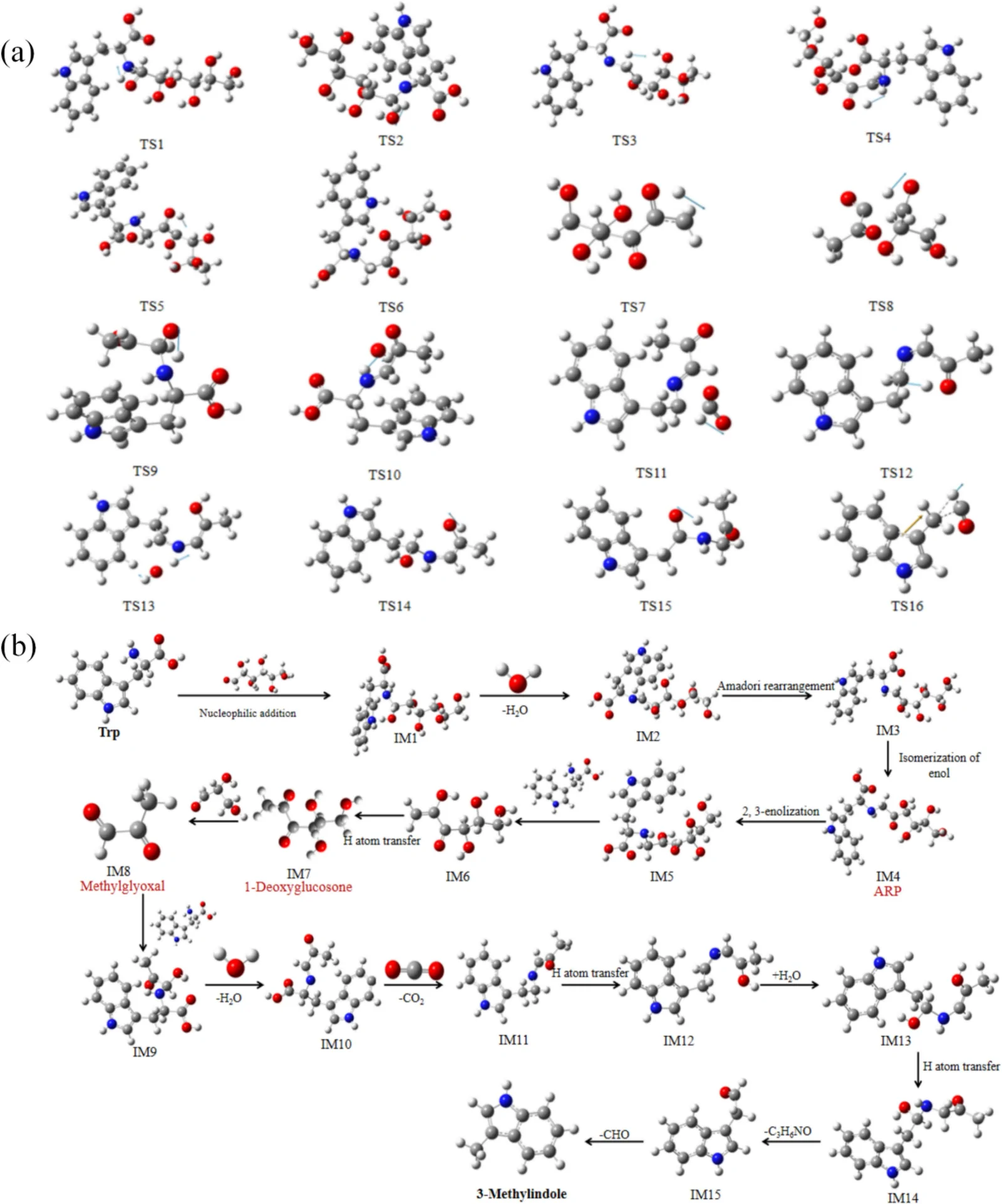
a. The optimized geometric configuration of the transition state in the reaction process. b. Structure optimization of reactants, intermediates and products in 3-methylindole formation pathway.

During the search for transition state structures, the program has already optimized the configurations of the transition states. Additionally, full structural optimization and frequency calculations at the B3LYP/6-311G+(d,p) level were performed for all reactants, intermediates, and products along the pathway, yielding their most stable configurations (Fig. 4b). All optimized structures exhibited zero imaginary frequencies, confirming they represent local minima on the potential energy surface. The optimization process involves identifying the lowest-energy, most stable structure to facilitate subsequent computational analysis. It also provides a reliable geometric configuration for subsequent calculation and analysis. Building upon the intricate reaction networks revealed by ReaxFF molecular dynamics simulations, density functional theory methods further performed high-precision optimisation and energy calculations on stationary points along key pathways, thereby elucidating the thermodynamic and kinetic parameters governing the dominant reaction channels.

### 3.5 Energy barrier changes during the formation of 3-methylindole

After performing the above calculations, the program determines the energy of each structure throughout the reaction process, ultimately yielding the overall energy change for the entire reaction, The energy profile for the entire 16-step 3-methylindole formation pathway was calculated (Fig. 5). The process involves overcoming a series of energy barriers. First, tryptophan and glucose undergo a nucleophilic addition reaction via TS1, crossing an energy barrier of 45.14 kcal/mol. The amino group of tryptophan attacks the carbonyl group of glucose to form IM1. Subsequently, IM1 undergoes TS2, crossing an energy barrier of 130.25 kcal/mol, and loses one molecule of H₂O to form IM2. IM2 undergoes an Amadori rearrangement, where the hydrogen from glucose's C1 position transfers to N, forming IM3 via TS3 with an energy barrier of 223.29 kcal/mol. IM3 undergoes enol isomerization, then undergoes TS5 to generate IM5 with an energy barrier of 188.33 kcal/mol. During this step, the C—N bond in IM5 breaks while a hydrogen atom transfers, leading to TS6 with an energy barrier of 431.08 kcal/mol to form IM6. IM6 undergoes hydrogen transfer via TS7 with an energy barrier of 432.57 kcal/mol to yield IM7 (1-deoxythreose). IM7 loses one molecule of C_3_H_6_O_3_, crossing TS8 with an energy barrier of 190.05 kcal/mol to form the α-diketone methylal. Subsequently, methylal undergoes Strecker degradation with tryptophan to yield indolealdehyde. First, the amino group of tryptophan attacks the carbonyl group of methylglyoxal. Both undergo TS9, crossing an energy barrier of 151.4 kcal/mol to form IM9. IM9, IM10, and IM11 all undergo intermolecular hydrogen transfer, respectively crossing TS10, TS11, and TS12 with energy barriers of 360.37, 360.75, and 124.8 kcal/mol to form IM10, IM11, and IM12, respectively. IM12 reacts with one molecule of H_2_O, which cleaves into OH and a free H radical that transfers to IM12. Crossing TS13 with an energy barrier of 278.01 kcal/mol yields IM13. IM13 undergoes H transfer, and then crosses TS14 with an energy barrier of 315.59 kcal/mol to form IM14, the precursor to indole-3-acetaldehyde. Concurrently with the cleavage of the C—N bond in IM14, hydrogen transfer occurs. Crossing TS15 with an energy barrier of 291.43 kcal/mol yields indole-3-acetaldehyde. Finally, indole-3-acetaldehyde loses one CO molecule. Crossing TS16 with an energy of 241.05 kcal/mol yields the final product, 3-methylindole. With the highest single barrier identified for the step involving TS7 (432.57 kcal/mol). Key energy-determining steps included the initial nucleophilic addition (TS1, 45.14 kcal/mol), the Amadori rearrangement (TS3, 223.29 kcal/mol), and the final decarbonylation from indole-3-acetaldehyde (TS16, 197.74 kcal/mol).

**Fig. 5:**
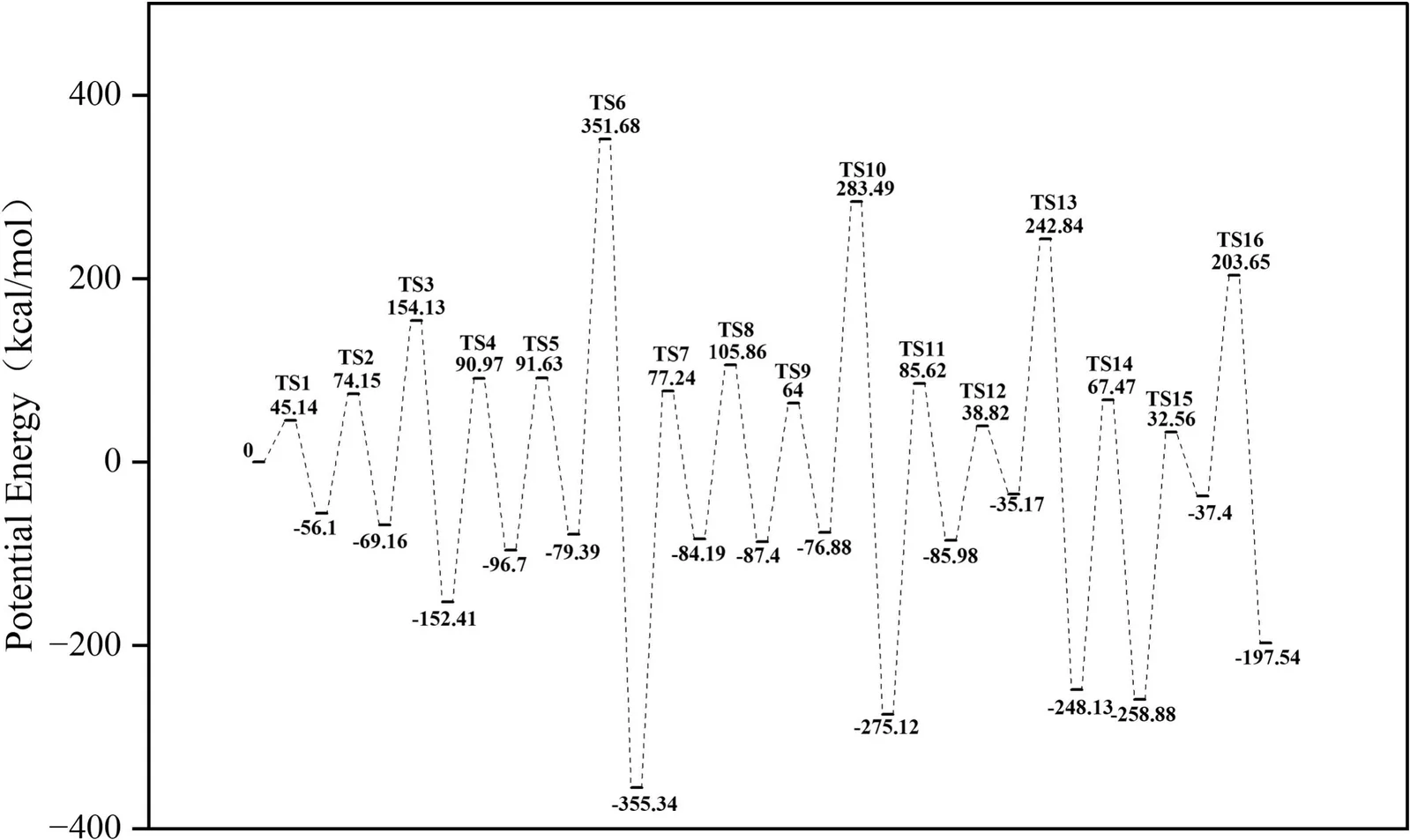
Energy barrier evolution during the formation of 3-methylindole.

Throughout this entire system, 15 reaction steps were newly identified. During the transformation from reactants to products, the energy of the transition state represents the highest energy point in the entire process. The complete energy landscape underscores the complexity and multi-stage nature of the reaction, with the transition state energies representing the critical kinetic bottlenecks throughout the pathway. Overall, the energy profile from reactants to final products exhibits a net increase, revealing an endothermic feature for the overall transformation.

### 3.6 Py-GC/MS analysis

ReaxFF MD and DFT were combined to investigate the formation pathway of 3-methylindole. To complement the computational predictions with experimental observations, Py-GC/MS analysis was performed. This technique detected key intermediates that are structurally consistent with those predicted by the simulations, including methylglyoxal and indole-3-acetaldehyde, as well as the final product, 3-methylindole. It should be noted that although the pyrolysis temperature (550 °C) differs substantially from the actual Maillard reaction conditions (120–140 °C), Py-GC/MS in this context functions as a structural fragment release tool—analogous to the EI source in mass spectrometry—to release characteristic structural units from complex samples, thereby assisting in the identification of fragment-level features of the intermediates. The integrated pathway, incorporating these experimentally detected intermediates and the calculated transition states, is reconstructed in Fig. 6a.

**Fig. 6:**
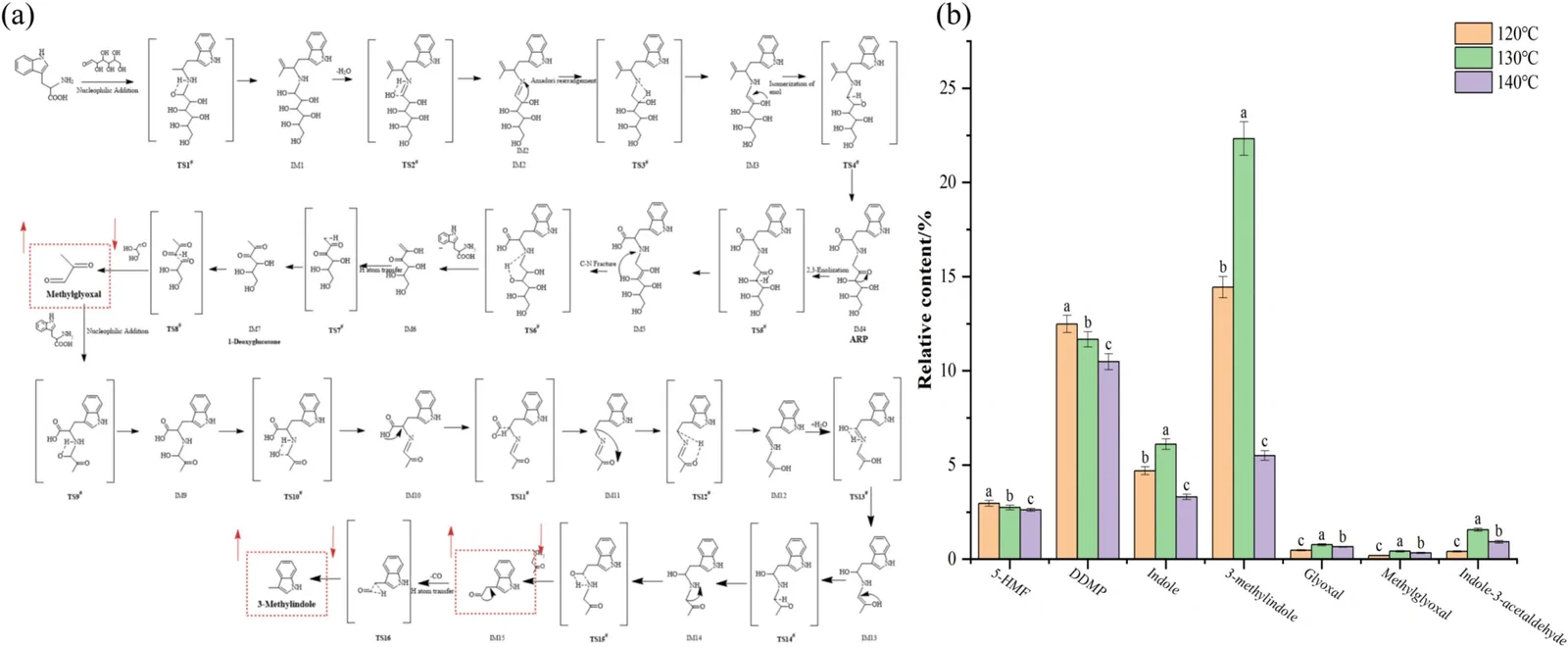
a. The trend of the key intermediates in the 3-methylindole formation pathway with temperature was obtained by combining Reax FF and DFT methods. b. The relative content changes of key intermediates and products in Maillard reaction.

The concentration trends of these key compounds with temperature provided supportive kinetic evidence for the proposed pathway. As shown in Fig. 6b, the concentration of methylglyoxal exhibited a trend of initial increase followed by a decrease at the highest temperature (140 °C), consistent with its expected behavior as a reactive intermediate. A similar trend was observed for indole-3-acetaldehyde. This may be attributed to the accelerated Maillard reaction progression at higher temperatures, thereby promoting methylglyoxal formation (Cai et al., 2024). Indole-3-acetaldehyde, a key precursor in the 3-methylindole pathway, exhibited a temperature-dependent concentration trend of initial increase followed by decrease, which is consistent with the GC-O-MS results. This non-linear behavior aligns with the ReaxFF MD results, suggesting that at elevated temperatures, increased molecular collision frequency may lead to the further degradation of these intermediates into smaller molecules, thereby reducing their steady-state concentrations. Collectively, these results are consistent with the notion that elevated temperatures can appropriately promote the Strecker degradation reaction between tryptophan and glucose, ultimately leading to the formation of 3-methylindole.

Collectively, the Py-GC/MS experiments detected key intermediates that are structurally consistent with those identified in the ReaxFF MD and DFT simulations. Although the pyrolysis conditions differ from the actual Maillard reaction conditions, these results indicate that the proposed pathway is chemically plausible, particularly in terms of the sequential appearance of methylglyoxal and indole-3-acetaldehyde as precursor fragments. The intermediate “snapshot” provided by Py-GC/MS shows consistency with the dynamic reaction trajectory revealed by multi-scale simulation. This consistency suggests that the integrated computational strategy has good applicability in analyzing complex Maillard reaction networks, and reinforces the credibility of the proposed 3-methylindole formation pathway.

### 3.7 DSC analysis

To further understand the thermal behavior of the reaction system from a macroscopic perspective, we carried out DSC analysis on tryptophan, glucose, and their mixture (Zhong et al., 2019). As shown in Fig. 7, the DSC curves indicate that the mixture exhibits a net endothermic feature, with major thermal events occurring between 83 °C and 156 °C. The peak around 83 °C can be attributed to the glass transition of glucose, while the peak near 156 °C likely corresponds to the early-stage reactions between tryptophan and glucose. Interestingly, the mixture shows a lower endothermic peak intensity in this region compared to the individual components, which may suggest intermolecular interactions between the two reactants. At higher temperatures, the DSC curve of the mixture becomes relatively flat. This observation is in line with the high energy barriers we calculated for the later reaction steps (e.g., TS7 at 432.57 kcal/mol and TS11 at 360.75 kcal/mol), implying that the reaction tends to slow down as the temperature rises. Overall, the endothermic direction observed in the DSC analysis matches well with the DFT-calculated energy profile, which shows a net increase from reactants to products. Although DSC reflects macroscopic heat flow and DFT deals with molecular-level energy barriers, their consistency in terms of endothermic direction provides supportive thermal evidence for the proposed reaction pathway (Berradi et al., 2026).

**Fig. 7:**
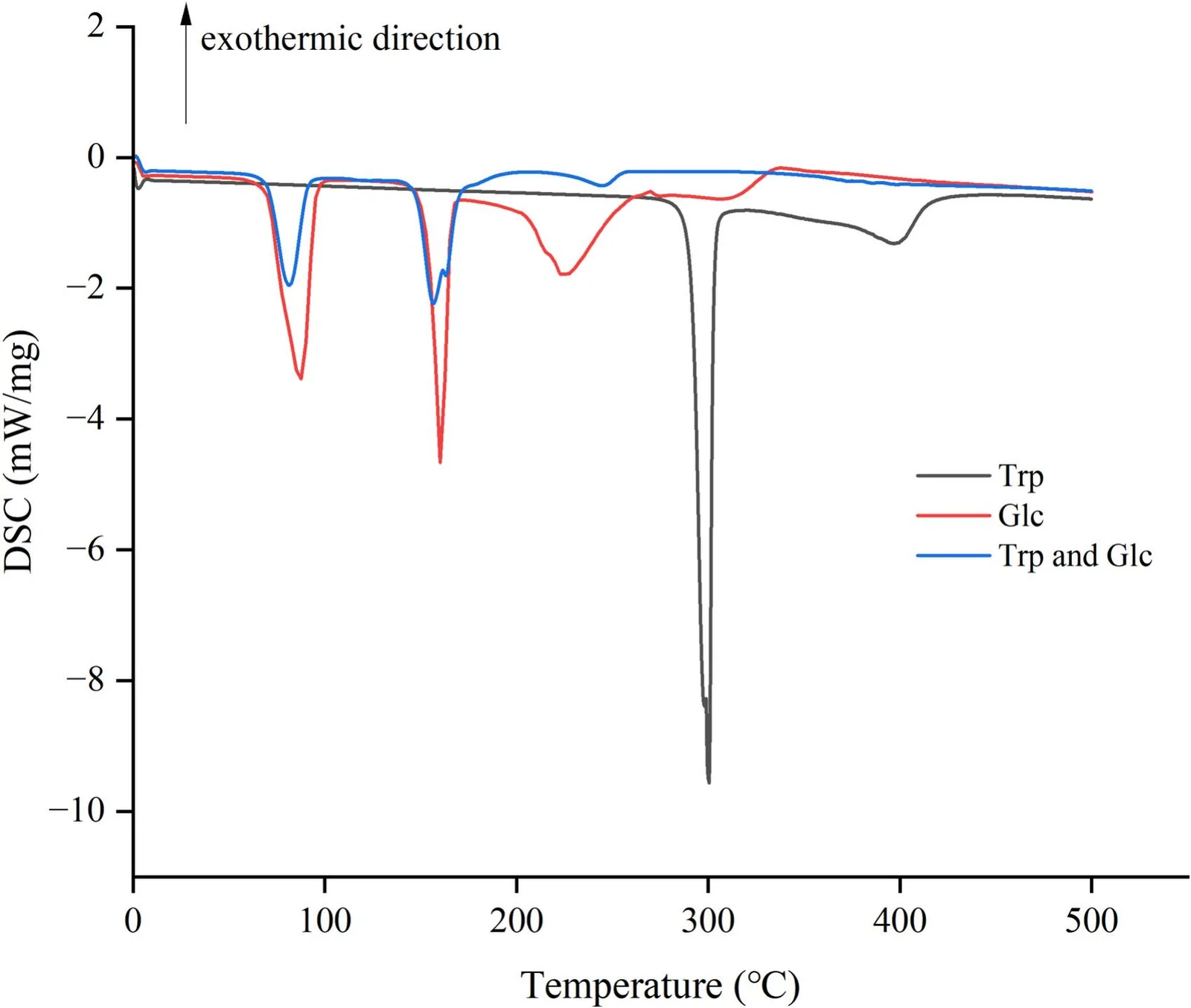
DSC curves of tryptophan, glucose and their mixture (heating rate 10 °C/min, N2 atmosphere).

## 4 Conclusions

In this study, the tryptophan-glucose Maillard model system was used to elucidate the formation mechanism of 3-methylindole by integrating aroma analysis, reaction molecular dynamics and quantum chemical calculations. Tryptophan and glucose were subjected to nucleophilic addition and Amadori rearrangement to form ARP, which was further subjected to C—N cleavage and hydrogen transfer to form methylglyoxal. The intermediate is involved in the Strecker degradation of tryptophan, and the generated indole-3-acetaldehyde is finally decarbonylated to 3-methylindole. This pathway connects the glucose-derived carbonyl chemistry with the conversion of the tryptophan side chain and clarifies the heat-induced molecular source of 3-methylindole. DFT calculations are located and verified 16 transition states. IRC analysis confirmed its connection with the corresponding reactants and products, and calculated the energy activation of each elementary reaction, indicating that the reaction was net endothermic. Two key intermediates, methylglyoxal and indole-3-acetaldehyde, were detected by Py-GC–MS, and their temperature-dependent concentration changes supported the proposed pathway. DSC analysis further shows that the overall reaction system exhibits a multi-stage net endothermic behavior, which is consistent with the DFT results. The combination of ReaxFF MD and DFT provides a coherent microscopic image and thermodynamic basis for elucidating the formation mechanism of this flavor compound, and also provides a methodological framework for the study of the mechanism of volatile compounds in complex thermally processed food systems. Future research can further quantify the relative contribution of competitive pathways under different processing conditions and combine isotope labeling or time-resolved spectroscopy to verify key intermediates.

## Founding sources

This work was supported by the 10.13039/501100001809National Natural Science Foundation of China (32401996), the 10.13039/501100012226Fundamental Research Funds for the Central Universities (10.13039/501100012490North Minzu University, 2025QNPY10), Natural Science Foundation of Ningxia Hui Autonomous Region (2026AAC050013), China Post doctoral Science Foundation (2024M761983), and Key research and development projects in Ningxia Hui Autonomous Region (2024BEE02034). Ningxia Hui Autonomous Region Technology Innovation Team for High-Quality Development of Characteristic Agricultural Products (2025CXTD002).

## CRediT authorship contribution statement

**Yuan-Yuan Yang:** Writing – review & editing, Writing – original draft. **Chao-Kun Wei:** Writing – review & editing, Supervision, Software, Funding acquisition, Conceptualization. **Xin Pan:** Validation, Software, Formal analysis. **An-Ran Zheng:** Supervision, Software, Funding acquisition. **Wen-Li Wang:** Supervision, Methodology. **Chun-Mei Yang:** Methodology, Data curation. **Yuan Liu:** Supervision, Funding acquisition.

## Declaration of competing interest

The authors declare that they have no known competing financial interests or personal relationships that could have appeared to influence the work reported in this paper.

## Data Availability

Data will be made available on request.

## References

[bb0005] Ahn J., Choi E., Lee K. (2025). Analysis of volatiles and α-dicarbonyl compounds in Maillard reaction products derived from 2′-fucosyllactose and amino acids. Food Research International.

[bb0010] Alcázar-Valle M., Lugo-Cervantes E., Mojica L., Morales-Hernández N., Reyes-Ramírez H., Enríquez-Vara J.N., García-Morales S. (2022). Bioactive compounds, antioxidant activity, and antinutritional content of legumes: A comparison between four *Phaseolus* species. Molecules.

[bb0015] Anjum V., Kadi A., Malinin A., Jha P., Potoroko I. (2026). Density functional theory study of fucoidan interactions with dairy metabolites for flavor and texture control in cheese. Food Bioscience.

[bb0020] Barrales-Martínez C., Durán R., Jaque P. (2024). Transition from synchronous to asynchronous mechanisms in 1,3-dipolar cycloadditions: A polarizability perspective. Journal of Molecular Modeling.

[bb0025] Berradi O., Berradi M., Elrhayam Y., Eddaoukhi A., Bouachri M., El Azzaoui J., El Youbi S.E. (2026). A new inorganic–organic hybrid crystal, (NH_3_(CH_2_)_6_)NH_3_SnCl_6_: Synthesis, crystal structure, vibrational, thermal, morphological properties, and DFT analysis. ACS Omega.

[bb0030] Cai Y., Pan X., Zhang D., Yuan L., Lao F., Wu J. (2024). The kinetic study of 2-acetyl-1-pyrroline accumulation in the model system: An insight into enhancing rice flavor through the Maillard reaction. Food Research International.

[bb0035] Chen X., Wu P., Wang S., Sun J., Chen H. (2025). Identification of key aroma-active compounds in commercial coffee using GC-O/AEDA and OAV analysis. Foods.

[bb0040] Cheng X., Jiang D., Cao B., Wang S., Li H., Barati B., Xia Z., Zhou J., Wang S. (2022). Study on ZSM-5 catalytic pyrolysis mechanism of cellulose based on the Py-GC/MS and the density functional theory. Combustion and Flame.

[bb0045] Cui H., Yu J., Zhai Y., Feng L., Chen P., Hayat K., Xu Y., Zhang X., Ho C. (2021). Formation and fate of Amadori rearrangement products in Maillard reaction. Trends in Food Science & Technology.

[bb0050] Deng S., Cui H., Hussain S., Hayat K., Liu W., Zhang X., Ho C. (2025). Promoted formation of pyrazines by targeted precursor addition to improve aroma of thermally processed methionine-glucose Amadori compound. Food Chemistry.

[bb0055] Ding C., He Y., Wang Z., Xiao X., Zhao Y., Wang Y., Chen X., Guo R., She Y., Wang B., Liu Z. (2025). The alleviating effects of tryptophan metabolite indole-3-propionic acid on glycolipid metabolism disorders and cognitive dysfunction in obese mice. Food Bioscience.

[bb0060] Dong S., Guo L., Xuan C., Zhu C., Han Z., Chen N., Wang X., Shen M., Wei X., Yang H., Zhang X., Liu M., Zhou S., Zhou L. (2026). Lactobacillus plantarum ameliorates intrahepatic cholestasis by modulating the gut microbiota and tryptophan metabolism. Food Research International.

[bb0065] Fiore A., Murray P.J. (2021). Tryptophan and indole metabolism in immune regulation. Current Opinion in Immunology.

[bb0070] Frøst M.B., Hartmann A., Petersen M.A., Duelund L., Mouritsen O.G. (2021). Odour-induced umami - olfactory contribution to umami taste in seaweed extracts (dashi) by sensory interactions. International Journal of Gastronomy and Food Science.

[bb0075] Gao P., Wang S., Feng B., Liu C., Wang Y., Dou S., Dong L. (2025). Volatile profiling from thermal decomposition of Amadori compounds in the alanine-glucose Maillard reaction: An DFT insight of 3-ethyl-2,5-dimethylpyrazine forming mechanism. Food Chemistry: X.

[bb0080] Grambow C.A., Pattanaik L., Green W.H. (2020). Reactants, products, and transition states of elementary chemical reactions based on quantum chemistry. Scientific Data.

[bb0085] Guan T., Fu S., Wu X., Yu H., Liu Y. (2024). Bioturbation effect of artificial inoculation on the flavor metabolites and bacterial communities in the Chinese Mao-tofu fermentation. Food Chemistry: X.

[bb0090] Guo Q., Xu S., Liu H., Liu M., Wang C., Qin Z., Wang X. (2022). Effects of roasting temperature and duration on color and flavor of a sesame oligosaccharide-protein complex in a Maillard reaction model. Food Chemistry: X.

[bb0095] Huang D., Lv J., Li X., Gong W., Tian J., Gao X., Qin X., Du G., Zhou Y. (2026). Lactobacillus johnsonii ameliorates CUMS-induced depressive-like behaviors by modulating tryptophan metabolism and intestinal barrier function. Food Bioscience.

[bb0100] Huang Z., Zeng Y., Sun Q., Zhang W., Wang S., Shen C., Shi B. (2022). Insights into the mechanism of flavor compound changes in strong flavor baijiu during storage by using the density functional theory and molecular dynamics simulation. Food Chemistry.

[bb0105] Lei L., Wang S., Zhao Z., Dou S., Zhang S., Wang Y., Dong L. (2024). Analysis of volatiles from the thermal decomposition of Amadori rearrangement products in the cysteine-glucose Maillard reaction and density functional theory study. Food Research International.

[bb0110] Li K., Wang J., Zhuang Y., Yuan G., Li Y., Zhu X. (2023). Glucose-histidine Heyns compound: Preparation, characterization and fragrance enhancement. Carbohydrate Research.

[bb0115] Liu J., Sun H., Chen L., Ding G. (2023). Shielding effects of aromatic (indole) ring for structural analysis. Journal of Natural Products.

[bb0125] Liu X.S., Liu J.B., Yang Z.M., Song H.L., Liu Y., Zou T.T. (2014). Aroma-active compounds in Jinhua ham produced with different fermentation periods. Molecules.

[bb0130] Liu Z., Wang S., Huang L., Zuo S., Ji T. (2026). Linkage cleavage mechanisms and product evolution in softwood lignin pyrolysis: ReaxFF reactive molecular dynamics simulations. Journal of the Energy Institute.

[bb0135] Liu Z., Wang X., Qin H., Chen Y., Xia L., Wang X., Lai Y., Li G. (2026). Research progress of the Maillard reaction process monitoring. Chinese Chemical Letters.

[bb0150] Pan X., Wei C., Wang W., Weng Y., Zheng A., Sun L., Yang C., Liu Y. (2026). Formation of 5-methyl-2-furanmethanol which is a caramel flavor compound in the glutamic acid-glucose Maillard reaction: A ReaxFF-DFT study. Current Research in Food Science.

[bb0155] Shakoor A., Zhang C., Xie J., Yang X. (2022). Maillard reaction chemistry in formation of critical intermediates and flavour compounds and their antioxidant properties. Food Chemistry.

[bb0160] Sun P., Huang Y., Chen S., Ma X., Yang Z., Wu J. (2024). Indole derivatives as agrochemicals: An overview. Chinese Chemical Letters.

[bb0165] Sun Z., Li H., Zhu W. (2023). Reactive molecular dynamics simulations on the decomposition process of 1,3,5-trinitro-1,3,5-triazine crystal under high temperatures and pressure. Journal of Molecular Modeling.

[bb0170] Tang H., Li P., Chen L., Ma J.K., Guo H.H., Huang X.C., Jiang L.W. (2022). The formation mechanisms of key flavor substances in stinky tofu brine based on metabolism of aromatic amino acids. Food Chemistry.

[bb0175] Tian H., Cheng J., Li L., Chen C., Ma X., Yu H., Yuan H. (2024). Microbial diversity and aroma profile variations in stinky tofu across different regions. Food Bioscience.

[bb0180] Tian Y., Chen Y., Shu C., Liu Y. (2026). Transformation mechanisms of nitrogen and oxygen in biomass hydrothermal bio-oil: Insights from ReaxFF-MD and thermodynamic-kinetic analysis. Fuel.

[bb0185] Wang B., Dou S., Wang S., Wang Y., Zhang S., Lin X., Chen Y., Ji C., Dai Y., Dong L. (2024). Mechanism of thermal oxidation into volatile compounds from (E)-4-decenal: A density functional theory study. Food Chemistry: X.

[bb0190] Wang J., Jiang X.Z., Luo K.H. (2023). Exploring reaction mechanism for ammonia/methane combustion via reactive molecular dynamics simulations. Fuel.

[bb0195] Wang T., Wang N., Wang M., Wang L., Shi Y., Du J., Yu D. (2023). Theoretical exploration and experimental regulation of the degradation of Δ9-tetrahydrocannabinol in hemp seed oil by density functional theory. Food Research International.

[bb0200] Wang X., Wei C., Wei D., Yang C., Liu Y. (2026). Impact of Amadori rearrangement products of cysteine-xylose-glutamic acid on meat flavor development. Food Research International.

[bb0205] Xiao L., Wang S., Wang Y., Wang B., Ji C., Lin X., Liang H., Zhang S., Xu X., Dong L. (2023). Density functional theory studies on the oleic acid thermal oxidation into volatile compounds. Food Chemistry: X.

[bb0215] Zeng J., Cao L., Chin C., Ren H., Zhang J.Z.H., Zhu T. (2020). ReacNetGenerator: An automatic reaction network generator for reactive molecular dynamics simulations. Physical Chemistry Chemical Physics.

[bb0220] Zhang D., Duan X., Sun H. (2023). Phospholipidomics and quantum chemistry calculation unravel the changes in phospholipid molecules of flaxseed oil during roasting. Food Chemistry.

[bb0225] Zhang T., Fang K., Ni H., Li T., Li L.J., Li Q.B., Chen F. (2020). Aroma enhancement of instant green tea infusion using β-glucosidase and β-xylosidase. Food Chemistry.

[bb0230] Zhang Y., Gu M., Yang S., Fan W., Lin H., Jin S., Wang P., Ye N. (2025). Dynamic aroma characteristics of jasmine tea scented with single-petal jasmine “Bijian”: A comparative study with traditional double-petal jasmine. Food Chemistry.

[bb0235] Zhao J., Bai X., Du J., Chen Y., Guo X., Zhang J., Gao L. (2025). Tryptophan metabolism: From physiological functions to key roles and therapeutic targets in cancer (review). Oncology Reports.

[bb0240] Zheng A., Ni Z., Liu D., An Y., Thakur K., Wei C. (2024). Mechanism of bioactive 3,3′-diindolylmethane formation in flaxseed meal-xylose-tryptophan maillard reaction. Food Bioscience.

[bb0245] Zhong C., Tan S., Langrish T. (2019). Redness generation via Maillard reactions of whey protein isolate (WPI) and ascorbic acid (vitamin C) in spray-dried powders. Journal of Food Engineering.

[bb0250] Zhou X., Wen L., Xiao J., Mo X., Wan P., Chen D. (2024). 2-Acetyl-1-pyrroline originated from Maillard reaction is the key odorant of cooked Lipu taro. International Journal of Gastronomy and Food Science.

[bb0255] Zhu J., Li X., Liu Z., Sun X., Zhao L., Shi Y., Zhou G., Rui Z., Lu G. (2022). Effect of biofriendly amino acids on methane hydrate decomposition: Insights from molecular dynamics simulations. Fuel.

[bb0260] Zou L., Qi Y., Shen L., Huang Y., Huang J., Xia Z., Fan M., Fan W., Chai G., Shi Q., Zhang Q., Yan C. (2024). The neural representations of valence transformation in indole processing. Cerebral Cortex.

