# Supplementary material for: Unraveling the formation mechanism of 3-methylindole from tryptophan in the glucose-mediated Maillard reaction: a combined ReaxFF MD, DFT, and experimental study
Source: Food Chem X. 2026 Oct 31;40:104719. doi: 10.1016/j.fochx.2026.104719 (PMC13760478; doi:10.1016/j.fochx.2026.104719)
Supplement: Supplementary file 1 [file mmc1.docx]

**Supplementary Materials**

**Unraveling the Formation Mechanism of 3-Methylindole from Tryptophan in the Glucose-Mediated Maillard Reaction: A Combined ReaxFF MD, DFT, and Experimental Study**

**Fig. S1. Intrinsic reaction coordinate verification analysis**

**.**

**Table S1. Identification results of volatile components in Tryptophan-Glucose Maillard reaction system by GC-O-MS**

| **Number** | **Substance category** | **Compound Name** | **CAS Number** | **Relative content^a^** | | | **RI^b^** |
| --- | --- | --- | --- | --- | --- | --- | --- |
|  |  |  |  | 120℃ | 130℃ | 140℃ |  |
| 1 | Aldehydes | Octanal | 124-13-0 | 0.37±0.01^b^ | 0.42±0.01^a^ | 0.29±0.03^b^ | 1005 |
| 2 |  | Nonanal | 124-19-6 | 1.24±0.03^b^ | 1.34±0.02^a^ | 1.27±0.02^b^ | 1106 |
| 3 |  | Decanal | 112-31-2 | 0.83±0.02^c^ | 1.24±0.02^a^ | 1.14±0.04^b^ | 1208 |
| 4 |  | 2-Decenal, (E)- | 3913-81-3 | 0.14±0.01^c^ | 0.36±0.03^a^ | 0.24±0.03^b^ | 1531 |
| 5 |  | Acetylformaldehyde | 78-98-8 | 0.34±0.02^c^ | 0.64±0.03^a^ | 0.46±0.05^b^ | 1550 |
| 6 |  | 5-Hydroxymethylfurfural | 67-47-0 | 0.12±0.02^c^ | 0.27±0.05^b^ | 0.58±0.06^a^ | 920 |
| 7 |  | Glyoxal | 107-22-2 | 0.1±0.01^c^ | 0.16±0.03^b^ | 0.38±0.07^a^ | 1458 |
| 8 |  | DL-Glyceraldehyde | 56-82-6 | 0.17±0.05^b^ | 0.28±0.05^a^ | 0.17±0.04^b^ | - |
| 9 | Ketones | 2-Cyclopenten-1-one, 3-ethyl-2-hydroxy- | 21835-01-8 | 1.15±0.03^c^ | 1.33±0.02^a^ | 1.24±0.23^b^ | 1821 |
| 10 |  | 3-Undecanone | 2216-87-7 | 0.07±0.02^a^ | 0.03±0.02^b^ | 0.03±0.02^b^ | 1582 |
| 11 |  | 2-Undecanone | 112-12-9 | 0.05±0.01^a^ | 0.05±0.02^a^ | 0.03±0.02^b^ | 1385 |
| 12 |  | 1-Dodecanol | 112-53-8 | 0.23±0.02^b^ | 0.21±0.03^b^ | 0.49±0.54^a^ | 1581 |
| 13 |  | 2-Propanone, 1-hydroxy- | 116-09-6 | 0.06±0.03^c^ | 0.15±0.03^b^ | 1.19±0.06^a^ | 972 |
| 14 |  | Butane-2,3-dione | 431-03-8 | 0.18±0.01^c^ | 0.25±0.03^b^ | 0.59±0.03^a^ | 1952 |
| 15 |  | 2-Furanmethanol | 98-00-0 | 0.06±0.03^c^ | 0.14±0.04^b^ | 0.46±0.05^a^ | 1678 |
| 16 | Furans | Furfural | 1998/1/1 | 0.25±0.01^b^ | 0.25±0.03^b^ | 0.84±0.06^a^ | 612 |
| 17 |  | 2-Furanmethanol, 5-methyl- | 3857-25-8 | 2.65±0.04^b^ | 2.85±0.02^a^ | 2.61±0.07^b^ | 1731 |
| 18 | Indoles | Indole | 120-72-9 | 1.68±0.04^c^ | 1.9±0.02^a^ | 1.8±0.06^b^ | 2451 |
| 19 |  | Indole, 3-methyl- | 83-34-1 | 2.53±0.07^c^ | 3.18±0.05^a^ | 2.88±0.05b | 2512 |
| 20 | Nitrogen-containing compounds | 2-Acetylpyrrole | 1072-83-9 | 0.24±0.04^c^ | 0.34±0.01^b^ | 0.5±0.07^a^ | 1981 |
| 21 |  | Pyrazine, 2,5-dimethyl- | 123-32-0 | 0.76±0.02^c^ | 0.97±0.05^b^ | 1.47±0.78^a^ | 1322 |
| 22 | Others | Hexanoic acid, ethyl ester | 123-66-0 | 0.25±0.01^b^ | 0.27±0.04^b^ | 0.35±0.1^a^ | 2381 |
| 23 |  | 1-Deoxy-D-threo-pentulose | 60299-43-6 | 0.14±0.02^c^ | 0.32±0^a^ | 0.29±0.02^b^ | - |
| 24 |  | Quinoline | 91-22-5 | 1.44±0.02^c^ | 1.56±0.04^b^ | 1.62±0.02^a^ | 1955 |
| 25 |  | Acetic acid | 64-19-7 | 0.61±0.04^c^ | 0.85±0.06^b^ | 0.91±0.05^a^ | 1448 |
| 26 |  | Heptadecane | 629-78-7 | 0.25±0.03^b^ | 0.27±0.03^a^ | 0.21±0.03^c^ | 302 |
| 27 |  | Heneicosane | 629-94-7 | 0.15±0.04^b^ | 0.13±0.02^c^ | 0.22±0.11^a^ | 403 |
| 28 |  | 2,4-Di-tert-butylphenol | 96-76-4 | 2.07±0.04^a^ | 1.87±0.05^c^ | 1.83±0.06^b^ | 2251 |

a Internal Standard Quantification: Add 2 μL of 1,2-dichlorobenzene (50 μg/mL in methanol) as an internal standard to each sample.Cv = Sv/Si × Ci,(where Cv represents the concentration of the volatile compound and Ci represents the concentration of the internal standard; Sv represents the peak area of the volatile compound and Si corresponds to the peak area of the internal standard).

b RI, the retention index obtained by GC-O-MS (column: DB-wax,30 m×250 μm，0.25 μm film thickness).
